# *Theileria annulata*: Its Propagation in Rabbits for the Attenuation of Piroplasms in Cross-Bred Calves

**DOI:** 10.3390/ani12070813

**Published:** 2022-03-23

**Authors:** Muhammad Sajid Ramzan, Muhammad Imran Rashid, Haroon Akbar, Muhammad Avais, Muhammad Suleman

**Affiliations:** 1 Department of Parasitology, University of Veterinary and Animal Sciences, Lahore 54200, Pakistan; drsajidramzan@gmail.com (M.S.R.); drharoonakbar@uvas.edu.pk (H.A.); 2 Department of Veterinary Medicine, Faculty of Veterinary Science, University of Veterinary and Animal Sciences, Lahore 54000, Pakistan; mavais@uvas.edu.pk; 3 Institute of Microbiology, University of Veterinary and Animal Sciences, Lahore 54000, Pakistan; muhammad.suleman@uvas.edu.pk

**Keywords:** *Theileria annulata*, propagation in rabbit, attenuation, calves

## Abstract

**Simple Summary:**

Ticks and tick-borne diseases (TTBDs) are major hindrances for the growth and development of the livestock industry. Among TTBDs, theileriosis has the most important disease-causing high morbidity, mortality, and productive and reproductive losses in cattle. These parasites are mainly present in tropical and sub-tropical areas of the world, where they primarily infect ruminants. The current study was primarily aimed to attenuate the *T. annulata* in rabbits and to check its severity in susceptible cross-bred calves. Moreover, a comparison between experimentally infected and tested calves was also conducted. The significant rise in temperature and the percentage of infected cells were observed in the rabbits’ post-inoculation. All the infected calves showed a significant rise in temperature, piroplasm parasitemia %age, and lymph node enlargement, while the inoculated calves showed a slight rise in temperature, no piroplasm, and no enlargement of lymph nodes during the whole trial.

**Abstract:**

Tropical theileriosis caused by the protozoan; *Theileria annulata* is a tick-borne disease (TBD) transmitted by ticks of genus *Hyalomma*; is clinically characterized by fever, anemia, and lymphadenopathy; and is responsible for heavy economic losses in terms of high morbidity and mortality rates with reduced production. Infected red blood cells of *T. annulata* were inoculated into rabbits intraperitoneally, and propagation of *T. annulata* has been investigated. The current study has shown an association between induced tropical theileriosis and variation of body temperature in rabbits. A significant rise in temperature (39.92 ± 0.33 °C) was seen on day 8 onwards, with the maximum temperature (40.27 ± 0.44 °C) on day 14 post-inoculation. In the current study, in vivo trials in susceptible cross-bred calves to investigate the attenuation and comparison with the infected group were also conducted. All the infected calves (*n* = 5) showed a significant rise in temperature (40.26 ± 0.05 °C) on day 10 onwards, with the maximum temperature (40.88 ± 0.05 °C) on day 16. The temperature of inoculated calves increased gradually post-inoculation, but the difference was not significant. A maximum parasitemia of 20% was observed in infected calves, but no piroplasm parasitemia was observed in inoculated calves. The prescapular lymph nodes of infected calves were enlarged, while the lymph nodes of inoculated calves remained normal throughout the trial. Analysis of clinical and parasitological responses of infected and inoculated calves showed a significant difference (*p* ≤ 0.05) in terms of temperature, parasitemia, and lymph node scoring between two groups. The current study was primarily aimed to attenuate *T. annulata* in rabbit and to check its virulence in susceptible calves. It is concluded that propagation of *Theileria annulata* in rabbits made it attenuated. Rabbit can be used as an in vivo model to weaken the virulence of *T. annulata*.

## 1. Introduction

Tropical theileriosis is an important disease of cattle, caused by the protozoan *T. annulata*, in tropical and sub-tropical countries, including North Africa, Europe, India, and China. It is a tick-borne disease (TBD) that is transmitted by ticks of genus *Hyalomma* and clinically characterized by fever, anemia, lymphadenopathy, depression, dyspnea, tachypnea, jaundice, pneumonia, abortion, and mortality [1,2,3]. The disease is economically very important as it causes heavy economic losses in terms of high morbidity and mortality rates with reduced production [4]. Approximately 250 million cattle have been reported to be at risk of tropical theileriosis globally [5]. Its prevalence up to 96.5% and 5.14% have been reported in June and September in Pakistan, respectively [6,7]. There is a greater than 70% mortality rate in susceptible pure-breeds of cattle, while less than 45% mortality in cross-breeds has been reported [8]. Likewise, other TBDs, prophylactic measures for control of tropical theileriosis, include eradication of tick vector and immunization programs [4]. Resistance has been developed due to continuous application of acaricides and anti-theilerial drugs, and due to food safety concerns, there is need to adopt alternate strategies for controlling tropical theileriosis [9,10,11,12].

Vaccinologists suggested that attenuation could take place in unnatural hosts by passages. Vaccines against rabies and polio have been developed by passaging in mice or chicken embryo [13,14]. Live attenuated schizont-based vaccines against theileriosis, babesiosis and anaplasmosis have been effectively used in Morocco, Israel, Iran, India and Tunisia [15,16,17]. These vaccines either involve attenuation based on repeated in vivo passages of parasites in splenectomized calves such as *Babesia bigemina* and *Babesia bovis* or in vitro culture as in *Theileria hirci* and *Theileria annulata*, respectively [18,19,20]. Another in vitro attenuated-based vaccine of Babesiosis has been developed and is currently being used in Argentina [21]. Live protozoal vaccines cause mild infection in the host based on stimulation of protective immunity [22].

Tropical theileriosis caused by *T. annulata* and East Coast fever (*T. parva*) can be effectively controlled and prevented by the use of live vaccines. Muguga cocktail vaccine, which is known as live sporozoite vaccine, is being used in many African countries for the control of East Coast fever [23]. A live attenuated vaccine is responsible for T-cell mediated immune responses by accurate processing of intracellular organisms and antigen presentation, in association with Major Histocompatibility Complex (MHC) class I and class II antigens. The responses of T-cells are considered to be critical in protection against intracellular pathogens, and use of live vaccine mimics the initiation of both adaptive and innate immune responses as occur in natural infection and hence are responsible for appropriate regulatory and inflammatory immune responses in the host [24].

Edmond Sergent’s team firstly initiated vaccination against theileriosis in North Africa. It was based on isolation of field strains with low virulence and maintained in vitro via needle passaging in calves [25,26]. Attenuation of the intracellular parasite at its macroschizont stage could be achieved by continuous in vitro passaging [27,28]. Prolonged in vitro passaging of infected schizont cell lines has resulted in attenuation, in terms of reduced virulence [17,29]. Live attenuated vaccines have provided sterile immunity against homologous strain exposure and partial protection against heterologous strain exposure [29,30,31]. In vitro attenuated cell lines of *T. annulata* have been shown to be virulent with persistence of fever, parasitemia, and schizont proliferation, while, owing to the existence of only sub-clinical infection mainly characterized by reduced fever, these cell lines would be considered avirulent [32,33]. Immunization of cattle with in vitro cultured virulent lines resulted in partial attenuation at 50th passages and became avirulent at 130th passage based on parasitological and clinico-hematological aspects [34].

In vivo attenuation of *T. sergenti* in mutant and immunocompromised mice has been achieved successfully. *T. sergenti* infection was induced rapidly in the blood circulation of mice with an intact spleen, when supplied with bovine red blood cells [35]. Proliferation of *T. orientalis* in immunodeficient mice has also been reported. The mice showed high parasitemia level when infection was given through intraperitoneal route and upon continuous supply of bovine Red Blood Cells (RBCs). These mice were used as laboratory infection model for *T. orientalis* to induce sporozoites stage in (*H. longicornis*) ticks [36]. In earlier studies, all immunodeficient mice were splenectomized to extend the life span period of bovine RBCs [36,37].

Cell culture protocols are expensive to carry out and preserve the cell lines in very low freezing temperature conditions. Moreover, it is difficult to maintain the attenuated parasite in cells free of contamination, which is a long-term process that may take years to attenuate the parasite [17,38,39,40,41]. Hence, the current study primarily aimed to attenuate *T. annulata* in rabbit and to check its virulence in susceptible calves. The attenuation of *T. annulata* was not reported in a rabbit model experimentally.

## 2. Materials and Methods

### 2.1. Source of Parasite

We already isolated local strain of *T. annulata* from the blood of infected cross-bred Holstein cattle [7], and we maintained the infection in calves following the methodology of the previous studies [37,42], with slight modification. Blood samples were collected under sterile conditions from cattle calves showing signs of acute theileriosis in EDTA containing vacutainers. Briefly, the cattle calves were restrained with head elevated and jugular vein exposed. Antiseptic gauze (methylated spirit dipped) was used to remove dirt and debris. The jugular vein was engorged by applying pressure at the base of jugular groove. The needle was inserted in jugular vein, and blood was collected using sterile syringe. The blood was gently transferred to EDTA containing vacutainer. Thin blood smears were prepared from fresh blood and stained with Giemsa and observed under oil immersion lens at 1000× magnification of a compound microscope. The blood samples were further analyzed with PCR for confirmation of *T. annulata* with specific primers. These were taken from a total of 6 male healthy rabbits of the breed *Oryctolagus cuniculus* [43], age: 6–7 months, feed: standard palate feed, weight: 1350–1700 g approximately, and gender: male. The rabbits were quarantined in a separate place, and blood was collected from ear vein. The blood of rabbits was analyzed for blood parasites such as Babesia, Anaplasma, and Theileria, by microscopy and PCR analysis. The rabbits were declared free from any kind of infection under microscopic examination and PCR. Polymerase chain reaction (PCR) was performed using SimpliAmp Thermocycler (Catalog no. A24811; ThermoFisher Scientific (USA) according to the conditions of [44,45], with slight modification, and specific primers TannUNF = 5′-GGGAGCTACAGTCATAGGTGGT-3′, TannUNR = 5′-TCCTGCCATTGCCAAAAGTC-3′) [46] were used to obtain product size of 460 bp, for confirmation of *T. annulata*. The PCR mixture was prepared in a final volume of 20 µL. Initial denaturation was given at 95 °C for 5 min, and reaction was cycled for 35 times. Each cycle was started at denaturation at 95 °C for 30 s, annealing at 58 °C for 30 s, and extension step at 72 °C for 30 s, and a final elongation at 72 °C for 10 min. After that, these amplified DNA fragments were analyzed on 1.5% agarose gel electrophoresis.

### 2.2. Propagation of the Parasite in Rabbits

The rabbits were inoculated intraperitoneally with (3 × 10^6^ infected red blood cells) of acutely infected cattle calf following the methodology of [37], with slight modification of observing parasitemia after every 2 to 3 days. The blood contained piroplasms of *T. annulata*. The rabbits were monitored for their body temperature daily. The rabbits showed clinical signs of infection. Blood samples were collected after every 2 days up to one month for microscopic examination, for parasitemia estimation, and for PCR analysis. For PCR analysis, blood was drawn from rabbits on 8th day post infection.

### 2.3. Inoculation of the Parasite in Calves

The blood containing attenuated protozoan parasite was drawn from saphenous vein of these rabbits for inoculation into healthy calves. Ten cross-bred calves (4–8 months old) were screened for any infection and confirmed through microscopy and PCR analysis. The calves were reared in experimental station at University of Veterinary and Animal Sciences (UVAS), Lahore under natural climatic conditions and received ad libitum feed and water. These calves were divided into two groups—Infected and Healthy—based on their screening. Each of these two groups contained calves (*n* = 5). The calves in healthy group were inoculated with live (5 × 10^6^) rabbit-propagated-*Theileria*-infected RBCs subcutaneously, while calves in infected group (*n* = 5) were reared separately. All the calves in both groups were monitored for temperature, *Theileria* presence, and lymph node scoring daily, along with parasitaemia at every 2 day interval post-inoculation up to 30 days following the methodology of the previous studies [47,48], with slight modification. The increase in pre-scapular lymph nodes size was recorded by palpation and scored from 0 to 3 on a scale, with 0 = normal size; 1 = 2× normal size (mild response); 2 = 4× normal size (moderate response); and 3 = 10× normal size (grossly enlarged), as compared to size of lymph nodes on day 0 [49]. The parasitemia was monitored by thin smear slides’ preparation and counting of infected red blood cells.

### 2.4. Experimental Design and Statistical Analysis

Two groups of each rabbits and calves were made containing 6 and 5 in numbers, respectively. The results obtained from infected and inoculated calves were statistically analyzed by using *t*-test and analysis of variance (ANOVA). GraphPad Prism version 6 software (GraphPad Software 7825 Fay Avenue, Suite 230, La Jolla, CA, USA) was used for the statistical analysis of data. An unpaired *t*-test was used to compare the mean between two independent groups, and a two-way repeated measure ANOVA was performed to compare the mean differences between infected and inoculated calves.

## 3. Results

### 3.1. The Effect of Theileriosis on Body Temperature and %Age Piroplasm Parasitemia in Rabbits

This study has shown an association of induced theileriosis with variation of body temperature in rabbits. A significant rise in temperature (39.92 ± 0.33 °C) was seen on day 8 onwards, with the maximum temperature (40.27 ± 0.44 °C) on day 14 post-inoculation. One animal died on day 19 post-inoculation. Necropsy lesions included distended bladder, hard and pale liver, hemorrhages on liver, heart filled with clotted blood, hemorrhagic crop, balloon-shaped intestine filled with liquid excreta, normal kidneys but discoloration, and lungs and trachea filled with pus. Theileriosis was the cause of death.

**Figure 1:**
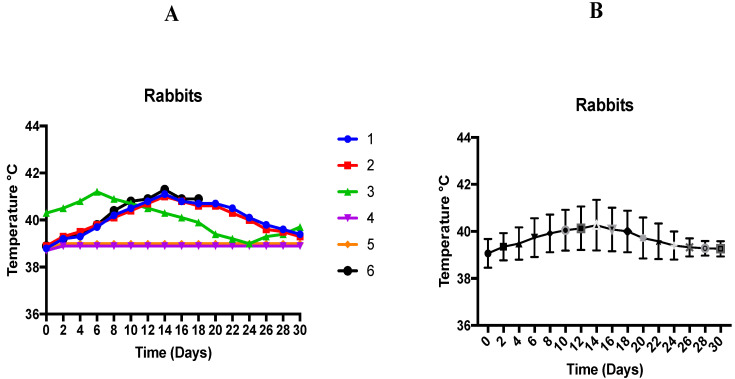
(**A**) Effect of temperature change on individual rabbit experimentally infected with *T. annulata*. (**B**) Temperature responses of rabbits following infection with *T. annulata*. Results are shown as mean °C ± SD.

The effect of temperature change on individual rabbit experimentally infected with *T. annulata* is shown in Figure 1A,B. The temperature of 4 rabbits was seen to rise from day 8 onwards to day 18 post-inoculation. Maximum temperature (41.3 °C) of one rabbit was observed on day 14 (this rabbit died on day 19 post-inoculation). The temperature of two rabbits remained normal, and both were clinically healthy during the whole trial.

**Figure 2:**
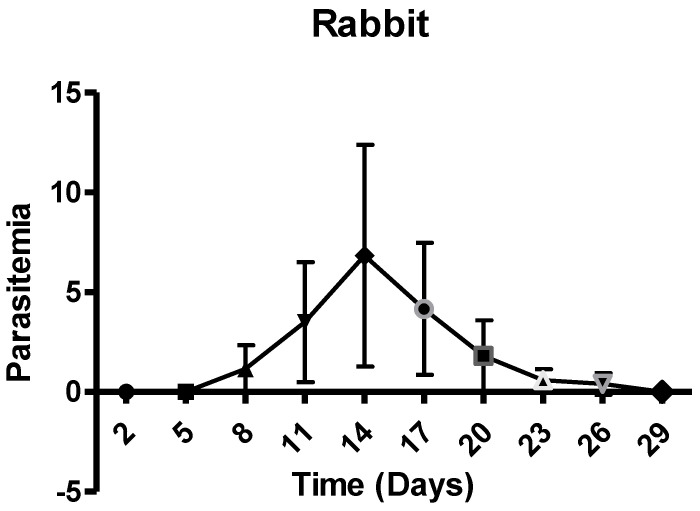
Post-infection %age piroplasm parasitemia of rabbits with standard deviation.

The results of %age piroplasm parasitemia are shown in Figure 2. The piroplasm parasitemia of rabbits appeared on 8th day post-inoculation and increased to 13% on day 14 during the trial. The parasitemia was not detectable after day 29 post inoculation.

#### Persistence of Infection in Rabbits

**Figure 3:**
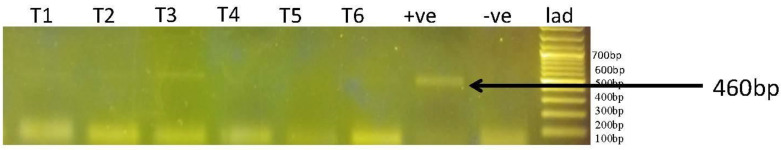
Results of PCR for infection expression in rabbits. T1 to T6 are samples from Rabbits, +ve is the positive control [46], and -ve control is without DNA.

Rabbits T1, T2, T3, and T6 showing light bands on PCR reveal the persistence of theileriosis on 8th day post-infection, respectively as shown in Figure 3.

### 3.2. The Effect of Theileriosis on Body Temperature, Parasitemia, Lymph Node Scoring, and Mortality in Experimental Calves

#### Infected and Inoculated Cattle Calves

(A) Temperature

**Figure 4:**
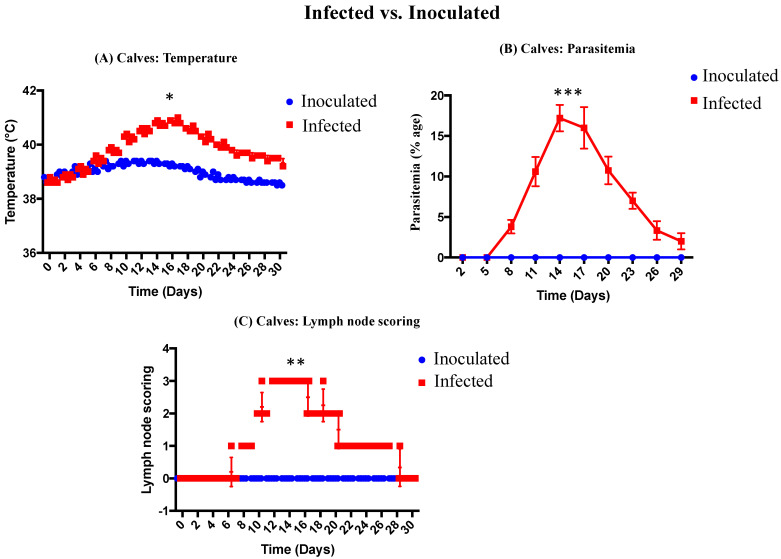
(**A**) Temperature of infected and inoculated calves. (**B**) Piroplasm parasitemia (%age) of infected and inoculated calves. (**C**) Lymph node scoring of infected and inoculated calves. Significant difference between infected and inoculated cattle calves at *p* ≤ 0.05. * denoted to ≤ 0.05, ** denoted to ≤ 0.005 and *** denoted to ≤ 0.0005.

All the infected calves (*n* = 5) showed significant rise in temperature (40.26 ± 0.05 °C) on day 10 onwards, with the maximum temperature (40.88 ± 0.05 °C) on day 16. One animal died on day 16 and one on day 22. The clinical signs observed during the trial were prescapular lymph node enlargement, anemia, icterus, protrusion of eyeballs, and lacrimation. Presence of diarrhea and recumbency was only observed in 2 animals. Post-mortem lesions included enlargement of spleen and liver, ascites, ulcers in the abomasum, and subcutaneous hemorrhages. The temperature of inoculated calves increased gradually post-inoculation, but the difference was not significant. The results are summarized in Figure 4A.

(B) Parasitemia

All infected calves (*n* = 5) showed piroplasm parasitemia in Giemsa-stained blood smears from day 8 onwards. A maximum parasitemia of 20% was observed (Figure 4B). In inoculated calves, there was no piroplasm parasitemia observed as compared to infected group. The results of %age piroplasm parasitemia are shown in Figure 4B.

(C) Prescapular lymph node responses

The results of prescapular lymph nodes of infected and inoculated calves are shown in Figure 4C. The prescapular lymph nodes of infected calves were enlarged, while the lymph nodes of inoculated calves remained normal throughout the trial. There was significant difference in lymph node scoring between infected and inoculated cattle calves.

## 4. Discussion

In this study, we demonstrated the first successful propagation for the purpose of attenuation of *T. annulata* in an un-natural host (rabbit). In rabbit, parasitemia can be developed upon inoculation of infected red blood cells of *T. annulata*, intraperitoneally. In the present study, we observed that the primary site of parasite proliferation was circulating blood, and our observation was strengthened by the reports of the previous study. Hagiwara et al. (1993) reported that the parasite may move from peritoneal cavity into the circulating blood through lymphatic system because the peritoneal cavity of mice appeared to be an inappropriate place for the proliferation of parasite. There is some evidence that suggests that the circulating blood is the primary site of parasite proliferation, as previously reported for *T. sergenti* proliferation in Severe Combined Immune Deficiency (SCID) mouse [37]. In the study, albino rabbits with intact spleen were used to evaluate the propagation of *T. annulata* in rabbit and active proliferation of piroplasm was confirmed on 8th day post-inoculation. A previous study on the propagation of *Theileria orientalis* in SCID mouse model also demonstrated the active proliferation of piroplasm within the RBCs on day 7 post-infection. Multiple piroplasms were detected within the individual RBC, which indicates multiplication of piroplasm occurred through binary fission, which is the common way of multiplication among *T. annulata*, *T. equi*, and *Babesia* but not in *T. parva* [50].

The parasitemia in rabbits lasted for about 28 days, but according to previous study [36], parasitemia in splenectomized SCID mouse lasted for nearly 2 months, 88 days in a SCID mouse without spleen, and 39 days in a SCID mouse with intact spleen, which clearly describes the role of spleen in removal of an infection [37]. Moreover, 23% parasitemia in splenectomized SCID mouse, and 7.4–21.4% in the SCID mouse with intact spleen, has also been reported, and we observed a maximum of 13% parasitemia in rabbit with intact spleen. In our study, parasitemia level increased to 1–3% on day 8 in rabbit model, but in previous study, 1% parasitemia was observed on day 7 in splenectomized SCID mouse model, when inoculated intravenously [37]. In our experiment, we also evaluated the propagation of *T. annulata* in rabbit model and upon inoculation into cattle calves; it produces mild signs and ensures protective immune response from tropical theileriosis.

In the current study, the pathological changes and clinical signs of infected and inoculated cattle calves were observed. During this study, high fever in *T. annulata*-infected calves was recorded, which is similar to other studies in which high fever, and anemia, was reported in *Theileria* affected cattle [51,52]. The clinical signs observed during the present study were similar to those observed in the previous studies in cattle naturally infected with *T. annulata* [53,54,55,56]. In the current investigation, all the infected calves (*n* = 5) showed significant rise in temperature (40.26 ± 0.05 °C) on day 10 onwards, with the maximum temperature (40.88 ± 0.05 °C) on day 16. According to previous study, a significant rise in temperature (40.14 ± 0.35 °C) on day 12 onwards, and maximum temperature (40.39 ± 0.28 °C) on day 16, was recorded in *T. annulata* infected cross-bred calves [57]. A significant rise in piroplasm %age parasitaemia (10.6 ± 0.81%) was observed on day 11 onwards, with maximum parasitaemia (17.2 ± 0.73%) on day 14 post-inoculation. The gradual increase in the parasitemia (%age) was linked with a gradual increase in body temperature and mortality in cattle calves infected with *T. annulata*. Our results are in accordance with the earlier reports, in which a progressive increase in %age parasitemia with the rise in body temperature was observed in case of acute *T. annulata* infections in calves [47,57]. It is well documented that the macroschizont stage, which is pre-erythrocytic stage of *T. annulata*, is mainly responsible for pathology in infected animals. The cells infected with macroschizont that are not able to produce piroplasms result in the induction of disease symptoms and mortality in cattle before the detection of piroplasm-infected erythrocytes, as compared to the related apicomplexan *Plasmodium* [58,59]. We observed the enlargement of pre-scapular lymph nodes in all the infected cattle calves during the trial. The same observations were reported in previous studies [60,61]. In this study, both schizonts and piroplasms were detectable in the blood smears of infected calves on 8th day post-infection, while according to the reports of the previous study, both piroplasms and schizonts were observed on 14th day post-infection in cattle [60].

The temperature, parasitemia, and lymph nodes of inoculated calves were normal during the whole trial as compared to infected group in the this study. All the inoculated calves showed no clinical signs except slight rise in temperature, and schizonts and erythrocytic stages were absent in blood smear. Our findings are similar to the previous study; attenuation is considered to be completed when inoculated calves exhibit no clinical signs except slight rise in temperature, and absence of either schizonts or erythrocytic stages in smear [59]. There was no pyrexia, no piroplasm parasitemia, no enlargement of lymph nodes, and no mortality in inoculated group. Our results are comparable to previous study, and according to Darghouth et al., (1996), there are four indicators used for loss of virulence: piroplasm parasitaemia, pyrexia, number of fatalities, and PCV [62]. Absence of clinical signs and fever in inoculated calves indicated that the rabbit-propagated-*Theileria*-infected RBCs were effective, safe, non-pathogenic, and suitable for use. Our findings are supported by the previous studies, in which it is well documented that the vaccine would be safe, non-pathogenic, and suitable for field use in the absence of fever and other clinical reactions in vaccinated animal [63,64,65]. The post-reactions of inoculated cattle calves were comparable to other attenuated cell lines reactions in animals. It is reported in some previous studies that the presence of only mild reactions or occasional piroplasm in few vaccinated animals, the cell line used for vaccination, is considered to be attenuated and that these mild reactions are due to live vaccine [32,63,66,67]. In the current study, there was no clinical case observed in inoculated calves during the whole trial. Our observations are also comparable to previous report in which Singh et al. (2001) reported that the vaccine was able to prevent the infection as no clinical cases but a lower number of piroplasm carrier animals were observed in vaccinated group [63].

## 5. Conclusions

In conclusion, we demonstrated the first successful propagation for the purpose of attenuation of *T. annulata* in the rabbits used as an unnatural host. A significant rise in temperature and parasitaemia were observed in rabbits upon the inoculation of infected red blood cells of *T. annulata*. Trials in susceptible calves did not show rise of temperature and parasitemia upon inoculation of rabbit-propagated-*Theileria*-infected RBCs. This study will be helpful for the development of live-attenuated vaccine against *T. annulata* in future.
